# Are non-slip socks really 'non-slip'? An analysis of slip resistance

**DOI:** 10.1186/1471-2318-9-39

**Published:** 2009-08-25

**Authors:** Satyan Chari, Terrence Haines, Paul Varghese, Alyssia Economidis

**Affiliations:** 1 Safety and Quality Unit, Royal Brisbane and Women's Hospital, Queensland Health, Queensland 4029, Australia; 2 Faculty of Medicine, Nursing and Health Sciences, Monash University, Victoria 3800, Australia; 3 Clinical Research, Southern Physiotherapy School, Monash University, Victoria 3168, Australia; 4 School of Health and Rehabilitation Sciences, The University of Queensland, Queensland 4072, Australia; 5 Allied Health Research Unit, Kingston Centre, Southern Health, Victoria 3192, Australia; 6 Geriatric Medicine, Geriatric and Rehabilitation Unit (GARU), Princess Alexandra Hospital, Queensland Health, Queensland 4102, Australia; 7 Early Assessment and Medical Unit, Internal Medicine Services, The Prince Charles Hospital, Queensland Health, Queensland 4032, Australia

## Abstract

**Background:**

Non-slip socks have been suggested as a means of preventing accidental falls due to slips. This study compared the relative slip resistance of commercially available non-slip socks with other foot conditions, namely bare feet, compression stockings and conventional socks, in order to determine any traction benefit.

**Methods:**

Phase one involved slip resistance testing of two commercially available non-slip socks and one compression-stocking sample through an independent blinded materials testing laboratory using a Wet Pendulum Test.

Phase two of the study involved in-situ testing among healthy adult subjects (n = 3). Subjects stood unsupported on a variable angle, inclined platform topped with hospital grade vinyl, in a range of foot conditions (bare feet, non-slip socks, conventional socks and compression stockings). Inclination was increased incrementally for each condition until slippage of any magnitude was detected. The platform angle was monitored using a spatial orientation tracking sensor and slippage point was recorded on video.

**Results:**

Phase one results generated through Wet Pendulum Test suggested that non-slip socks did not offer better traction than compression stockings. However, in phase two, slippage in compression stockings was detected at the lowest angles across all participants. Amongst the foot conditions tested, barefoot conditions produced the highest slip angles for all participants indicating that this foot condition provided the highest slip resistance.

**Conclusion:**

It is evident that bare feet provide better slip resistance than non-slip socks and therefore might represent a safer foot condition. This study did not explore whether traction provided by bare feet was comparable to 'optimal' footwear such as shoes. However, previous studies have associated barefoot mobilisation with increased falls. Therefore, it is suggested that all patients continue to be encouraged to mobilise in appropriate, well-fitting shoes whilst in hospital. Limitations of this study in relation to the testing method, participant group and sample size are discussed.

## Background

Falls continue to remain among the highest reported causes of unintended harm to elderly patients in hospital [1]. Several multifactorial interventions to prevent falls in hospitals have been investigated, and some have demonstrated effectiveness [2-5]. However, few have focussed on the role of footwear in the prevention of in-hospital falls. The 2005 Australian Falls Prevention Guidelines (Preventing Falls and Harm from Falls among Older People) recommend that patients wear well fitting, closed shoes with a flat heel while in hospital [6]. However, ensuring compliance with this recommendation is sometimes difficult due to patient cognitive impairment (confusion, delirium, dementia), lack of access to appropriate footwear, intentional non-compliance and other factors.

Concurrently, there has been a growing recognition of hospital morbidity and mortality due to venous-thromboembolism (VTE) [7]. The Australian and New Zealand Working Party on the Management and Prevention of Venous Thromboembolism suggest consideration of graduated compression stockings as an adjunct to pharmacological prophylaxis, in the management of patients at risk of VTE [8].

As patients can tend to mobilise without footwear while in hospital, there has been concern among clinicians that patients might be at an increased risk of falling due to the 'slippery' texture of most compression stocking products. As a result, the use of 'non-slip' socks over compression stockings has gained popularity as a strategy to improve under-foot traction. Non-slip socks (also referred to as anti-skid or treaded socks) are socks with a tread pattern provided on the sole, or ventral surface, for the purpose of improving traction. These socks appear to be a logical solution, however there is limited evidence regarding their effectiveness in improving traction and more importantly, their impact on patient safety.

One retrospective study evaluated falls rates for a 102 day period before and after implementation of a non-slip sock (treaded sock) intervention in a Special Care Unit at a nursing home [9]. In total, twenty one falls were recorded in 102 days prior to intervention and eighteen falls were recorded in the 102 days after intervention; a modest change that was not statistically significant. An eight-fold reduction in falls due to slips on urine was reported by the authors, who attributed this positive finding to the treaded sock intervention. However, there was a concurrent five-fold increase in falls where patients were 'found on the floor', which suggests that the intervention had little positive effect overall and that modified reporting might have been a factor as staff were not blinded to the intervention period.

This study aimed to establish the slip-resistance of non-slip socks relative to other foot conditions commonly encountered in hospital, in order to determine any traction benefit. Additionally, the data generated through this study would help inform decisions on further clinical research on non-slip socks as a falls prevention strategy.

## Methods

### Design

Ethics approval was sought and gained from the Princess Alexandra Hospital Human Research Ethics Committee. A two-phase study was designed. In phase one, two commercially available non-slip socks and one brand of compression stockings were tested for slip resistance through a blinded, independent materials testing laboratory (Commonwealth Scientific and Industrial Research Organisation's Manufacturing and Materials Technology Laboratory in Victoria). The samples were tested using a Pendulum Friction Test, also referred to as the Wet Pendulum Test [10,11]. The Wet Pendulum Test was selected instead of the alternative Inclined Ramp Test described in AS/NZS 4586:2004 [10], as it provides a continuous (rather than ordinal) measure, and is therefore more sensitive to small differences in slip resistance. Phase two involved in-situ testing of slip resistance of non-slip socks and other foot conditions among healthy adults. Phase two testing was analogous to the Inclined Ramp Test prescribed by the Australian Standard [10]. However, the method of testing followed in phase two, arguably provides a better approximation of standard hospital flooring in a dry state whilst also allowing for a continuous measure of slip resistance.

### Phase One

The Wet Pendulum Test was carried out at the Commonwealth Scientific and Industrial Research Organisation's (CSIRO) Materials, Surfaces and Finishes laboratory at Highett, Victoria. The testers were blinded to brand and manufacturer details of samples provided (all tags and identifiers removed), but not to product function. Samples were labelled A (compression stocking), B and C (non-slip socks) respectively.

### Apparatus and Procedure

**Figure 1:**
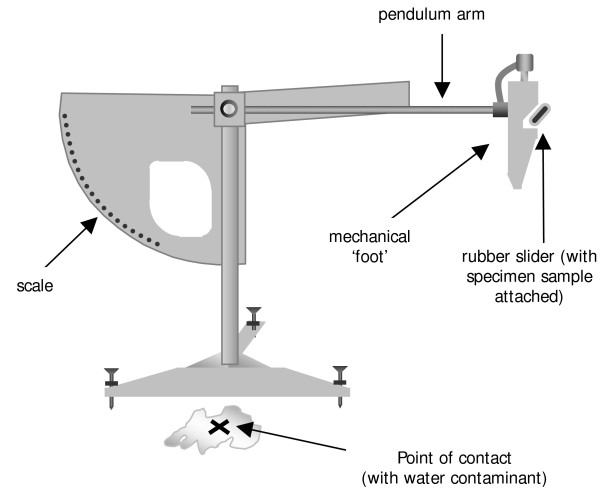
**Phase one testing apparatus: Wet Pendulum Test**.

The Wet Pendulum Test was completed in accordance with AS/NZS 4586:2004 [10] using a calibrated Munro-Stanley Pendulum Friction Tester. The Wet Pendulum Test is a test designed to simulate the mechanics of a person slipping on a wet surface. The terminal end of the pendulum arm has a mechanical foot with a spring-loaded rubber slider attached, to simulate the heel of the foot (Figure 1). The floor surface is saturated with deionised water prior to testing to simulate the presence of a fluid contaminant. The test is set up with the apparatus level to the floor and the length of the pendulum arm adjusted such that the rubber slider 'kicks' through when released, contacting the floor surface momentarily. On 'kicking' through, a loss of momentum occurs due to the friction generated at the point of contact. The amount of friction is dependant on the slip resistance characteristics of the floor and 'heel' surfaces. This loss in momentum causes a proportionate reduction in the arc described by the pendulum which is measured on an inverse scale affixed to the tester. The scale provides a British Pendulum Number (BPN) which is the unit of measurement for this test. A higher BPN indicates higher slip resistance. A detailed description of the Pendulum Friction Testing protocol is available as an appendix to the AS/NZS 4586:2004 [10].

The Wet Pendulum Test is normally used to test different floor surfaces for slip resistance while the rubber slider is conditioned before testing to provide a constant slip resistance. However, for the purpose of this study the conditions were reversed, by keeping the floor surface constant (2.0 mm thickness hospital graded vinyl) and varying 'foot' conditions by draping the test samples over the slider. Samples were adhered to the rubber slider using double-sided adhesive tape to eliminate bunching of the material on contact. Five different specimens cut from the ventral surface of the samples were tested three times each, producing fifteen readings per sample. Testing was performed at an ambient temperature of 23°C (prescribed testing temperature range).

### Phase Two

**Figure 2:**
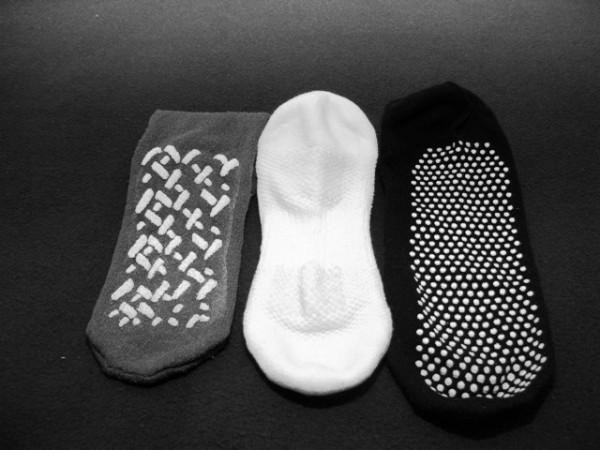
**Non-slip sock samples**.

Phase two of the study was conducted at the Princess Alexandra Hospital's Physiotherapy Gait Laboratory. A convenience sample of three brands of non-slip socks were tested (Figure 2). All of the non-slip sock products tested are commercially available in Australia and marketed for use with hospital patients. The non-slip socks brands tested in phase one (samples B and C) were included in phase two. An additional non-slip sock product was included in phase two as the investigators only became aware of the existence of this product following the completion of phase one.

Additional foot conditions tested in phase two were conventional socks (worn by the participants on the day of testing), bare feet and a compression stocking product currently used by facilities in Queensland Health (also tested in phase one).

Two male (Participant B and C) and one female participant (Participant A) were included in phase two of this study. Written informed consent was secured from participants prior to commencement. Participant A was 173 cm in height, weighed 65 kg and wore Australian size 8 women's footwear. Participant B was 182 cm in height, weighed 105 kg and wore Australian size 11.5 men's footwear. Participant C was 186 cm in height, weighed 85 kg and wore Australian size 12 men's footwear. All participants were aged between 29 and 31 years on the day of testing.

### Apparatus and Procedure

**Figure 3:**
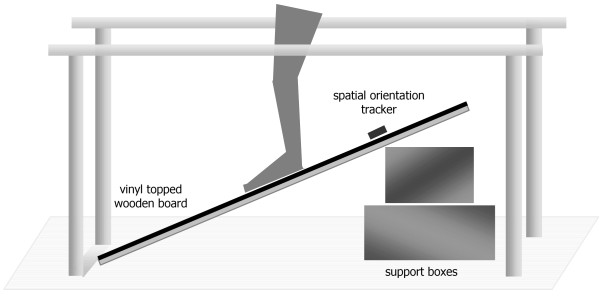
**Phase one testing apparatus: Inclined Ramp Test**.

The surface of the ramp was constructed by mounting a 900 mm × 600 mm panel of 2.0 mm thickness hospital grade vinyl (AS/NZS 2055.1:1985) [12], on to a rigid wooden board as per manufacturer's instructions. The ramp was bracketed on one end and the angle of inclination was adjusted by shifting support blocks forwards or backwards. The ramp was positioned in between a set of mobile parallel bars (Figure 3). An Intersense InteriaCube^® ^inertial orientation reference system was used to accurately measure the inclination of the ramp. The sensor was taped to the surface of the ramp such that the 'pitch' reading provided the angle of inclination. Data was monitored on a laptop in real time. The testing procedure was recorded on video to enable verification of manually collected data prior to transcription to a spreadsheet for analysis.

Once the angle of inclination was set (with an error tolerance of 0.2°), participants were asked to stand on the ramp and attempt to maintain an erect posture unsupported for a minimum of three seconds. If successful, the angle of the ramp was increased in one degree increments and the test was repeated until a slippage point was noted. Once slippage occurred, testing of that particular foot condition for the participant was complete. This procedure was repeated for every foot condition with each participant (non-slip socks, compression stockings, bare feet, and standard socks). Where multiple sizes of a non-slip sock product were available, these were also tested. The ramp surface was wiped down periodically to reduce contaminant build-up during testing.

## Results

### Phase one

**Figure 4:**
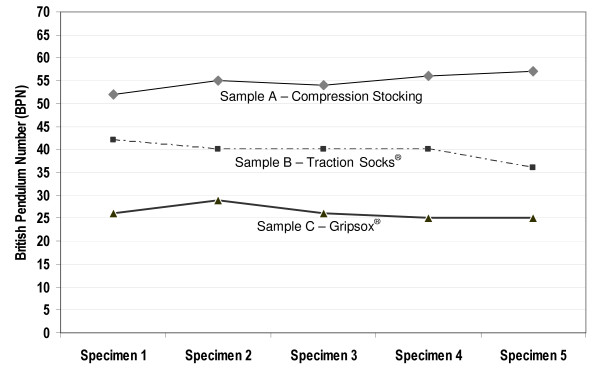
**Phase one wet pendulum test results**.

The compression stocking sample achieved the highest mean British Pendulum Number (55) followed by the two non slip sock samples B (40) and C (26), indicating that the compression stocking demonstrated the highest slip resistance in this testing condition (Figure 4). There was little variation in results from the five different areas of sole tested.

### Phase two

**Figure 5:**
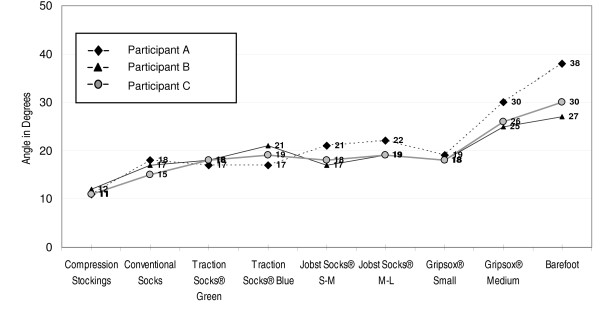
**Phase two test results: slippage points**.

Results of phase two testing demonstrated a relatively consistent slippage pattern across all three participants (Figure 5). However, in contrast to the Wet Pendulum Test in phase one, all participants slipped at the lowest angles while wearing compression stockings (12°,11° and11° respectively). Performance in conventional socks was relatively better, with slippage at 18°, 17° and 16° respectively. Performance in non-slip socks was variable, with slippage occurring in some products at angles comparable to conventional socks. Other non-slip socks performed better with traction maintained up to 30° in the case of one participant for a specific size of a non-slip sock brand. Different sizes within the same product also varied in performance (slippage at 19° for the 'small' sized sock and 30° for the 'medium' sized sock of the same brand in one participant). Barefoot conditions consistently resulted in the highest levels of traction across all participants with slippage at 38°, 27° and 30° respectively.

## Discussion

Previous studies have associated mobilisation in foot conditions other than shoes (such as slippers, sandals, socks, bare-feet and other 'non-ideal' foot conditions) with an increased risk of falling [13-15]. The poorer relative performance of non-slip socks compared to barefoot conditions (a 'non-ideal' foot condition) in our study suggest that non-slip socks do not represent an adequate alternative to well-fitting rubber soled footwear or even to mobilisation in bare feet.

Additionally, ensuring that non-slip socks are being worn appropriately (with tread pattern aligned with the sole of the foot) would most likely require periodic checks by clinical staff, especially if provided to patients with cognitive impairment. Aside from the resource implications, poorly fitted socks or socks that are mis-aligned could constitute a trip or slip hazard for patients. It is suggested that these risks might outweigh the clinical benefits of marginally improved underfoot traction over compression stockings.

All non-slip sock products tested in this study had a tread pattern on the ventral surface (sole) of the sock (Figure 2). This tread pattern is three-dimensional in nature resulting in a series of peaks (1–2 mm high) and troughs. In phase one, specimens cut from the three samples were adhered to the rubber slider using double sided tape. As the Wet Pendulum Test generates limited downward force during the 'kick' phase of the test, force at the point of contact is predominantly along the horizontal plane. As a result, the rubber slider does not press down on the troughs. Consequently, only the raised portion of the sock specimen would make contact with the floor, reducing what could be termed as the 'effective contact area' and therefore reducing slip resistance. This phenomenon is avoided in the compression stocking sample due to the absence of a tread pattern resulting in a level surface. In this case, the effective contact area would be equal to the size of the specimen adhered to the rubber slider.

When tested in-situ during phase two, the combined effect of participant weight and pliant characteristics of soft tissue of the foot are likely to force contact between the troughs of the non-slip sock and the floor thereby ensuring contact is made between the whole foot and floor across all testing conditions. This difference is proposed a plausible explanation for the apparent lack of congruence between phase one and phase two results.

Nagata, Watanabe, Inoue and Kim (2008) studied the validity of five different friction testing methods as an index of the risk of slipping with seventy subjects and concluded that of the five methods tested, the ramp test was the most reliable, and the pendulum tester the least reliable [16]. These results appear to validate the incongruence between results of two phases of our study.

There is also a possibility that the relative performance of non-slip socks and compression stocking is altered in the presence of a fluid contaminant. This hypothesis would require further investigation and if verifiable, has potential clinical implications when using non-slip socks with older persons having issues with bladder continence. Given previous findings that slips associated with standing in urine were reduced amongst nursing home residents wearing non-slip socks, one would have expected these socks to display greater slip resistance in the Wet Pendulum Test condition. However, this was not the case.

### Limitations

This study tested a convenience sample of non-slip socks, however it is recognised that there may be alternative products which perform differently.

This study tested non-slip sock performance on hospital grade vinyl which is the preferred floor covering as per AS 2055.1 [12]. However, it is possible that relative results might vary over other surfaces such as tile, polished concrete or carpet. Foot anatomy, biomechanics and skin characteristics of the relatively young and healthy participants in this study are also likely to be different to hospital patients who are older and frail. Some variation in performance across foot conditions could be expected with a sample of older hospital patients.

The testing protocol employed in phase two, although not standardised or previously validated, is very similar in method to the ramp test recommended in the Australian Standard [10]. However, the testing protocol still provides a reliable method to compare performance of various foot conditions within the same participant.

It needs to be acknowledged that the phase two ramp test collected slippage data with participants in a static standing position. It is conceivable that slippage characteristics, and therefore performance, of these foot conditions might vary during dynamic walking on a level surface.

The small number of participants can be considered a limitation of this study. However, we would like to highlight that the unit of analysis is not the individual participant but rather the results of the test in each foot condition, which is a product of the unique characteristics of the contact material (compression stocking, non-slip sock, conventional sock or skin), the fit of the particular sock (or compression stocking sample) to the participant's feet, and the weight of the participant. Additionally, we tested subjects with both large and small feet, as well as significant difference in weight, and found the results to follow a consistent pattern across all participants.

## Conclusion

Non-slip socks demonstrated poorer slip resistance than bare feet. It is therefore suggested that patients would be more likely to slip whilst mobilising in non-slip socks compared to bare feet. Non-slip socks offer marginal benefit in slip-resistance over compression stockings in dry conditions, however slip resistance of such products in the presence of fluid contaminants needs to be explored further. This study did not explore whether traction provided by bare feet was comparable to 'optimal' footwear such as shoes. However, previous studies have associated barefoot mobilisation with increased falls. It is therefore suggested that all patients continue to be encouraged to mobilise in appropriate, well-fitting shoes whilst in hospital.

## Competing interests

The authors declare that they have no competing interests.

## Authors' contributions

SC, TH, PV contributed to the development, conceptualization and design of the study. TH, SC and AE were responsible data collection and the conduct of Phase 2 testing. SC was responsible for data transcription, analysis, and preparation of the manuscript. TH supervised the data collection and provided assistance with data analyses and editing the final manuscript. All authors contributed to interpretation of results and read and approved the final draft of the manuscript.

## Pre-publication history

The pre-publication history for this paper can be accessed here:


